# The Impact of Surgical Approach on Mid-Term Clinical Outcomes Following Hemiarthroplasty for Femoral Neck Fractures: A Systematic Review and Meta-Analysis of Postero-Lateral Versus Direct Lateral Approaches

**DOI:** 10.3390/jcm14248846

**Published:** 2025-12-14

**Authors:** Gianmarco Marcello, Francesco Rosario Parisi, Lorenzo Alirio Diaz Balzani, Alessandro Del Monaco, Emanuele Zappalà, Giuseppe Francesco Papalia, Chiara Capperucci, Erika Albo, Augusto Ferrini, Biagio Zampogna, Rocco Papalia

**Affiliations:** 1 Operative Research Unit of Orthopaedic and Trauma Surgery, Fondazione Policlinico Universitario Campus Bio-Medico, Via Alvaro del Portillo, 200, 00128 Rome, Italyf.parisi@unicampus.it (F.R.P.); emanuele.zappala@unicampus.it (E.Z.); b.zampogna@policlinicocampus.it (B.Z.);; 2 Research Unit of Orthopaedic and Trauma Surgery, Department of Medicine and Surgery, Università Campus Bio-Medico di Roma, Via Alvaro del Portillo, 21, 00128 Rome, Italy; 3 Orthopaedics and Traumatology Unit, Ospedale dei Castelli, Via Nettunense, Km 11/5, 00040 Ariccia, Italy; 4 Oncological Orthopaedics Department, IFO-IRCCS Regina Elena National Cancer Institute, Via Elio Chianesi, 53, 00144 Rome, Italy; 5 BIOMORF Department of Biomedical, Dental, Morphological and Functional Images, University of Messina, A.O.U. Policlinico G. Martino, Via Consolare Valeria, 1, 98124 Messina, Italy

**Keywords:** hip hemiarthroplasty, postero-lateral, direct lateral, meta-analysis, dislocation, functional outcomes, complications

## Abstract

**Background**: Femoral neck fractures in the elderly often necessitate hemiarthroplasty, but the optimal surgical approach remains a highly debated topic. The postero-lateral and direct lateral approaches are commonly employed, each with benefits and drawbacks. Despite their widespread use, robust, long-term comparative studies on definitive outcomes, including pain, functional recovery, and complication rates, are notably lacking. This systematic review and meta-analysis aim to address this critical gap by meticulously comparing these approaches with long-term follow-up. **Methods**: A systematic literature search was performed, including only comparative studies with a minimum 1-year follow-up. A meta-analysis was performed for the primary outcome measures: operative time, dislocations, infections, perioperative fractures and reoperations. Secondary outcomes included a qualitative synthesis of patient-reported outcomes (quality of life, pain, and satisfaction). Methodological quality was assessed using RoB 2.0 for randomized controlled trials and MINORS criteria for cohort studies. **Results**: Our meta-analysis provides robust quantitative evidence. The direct lateral approach is associated with a significantly lower risk of post-operative dislocations (I^2^ = 58%; OR = 2.86, 95% CI: 2.53 to 3.22; *p* < 0.00001) and a significantly lower rate of reoperation (I^2^ = 0%; OR = 1.25, 95% CI: 1.12 to 1.40; *p* = 0.0001) compared to postero-lateral approach. Operative time, infection, and perioperative fracture rates were found to be statistically comparable. However, patient-reported outcomes yielded inconsistent results across studies, often becoming non-significant after adjusting for confounders. **Conclusions**: This meta-analysis shows that the direct lateral approach is associated with lower rates of dislocation and reoperation compared with the postero-lateral approach, while patient-reported outcomes remain variable across studies. Further high-quality comparative trials are needed to confirm these associations and guide surgical decision-making.

## 1. Introduction

Femoral neck fractures (FNFs) represent a significant public health concern, particularly among the elderly population, owing to their substantial contribution to morbidity, mortality, and long-term disability [1]. Hemiarthroplasty (HA) is widely accepted and frequently performed for displaced FNFs in this vulnerable demographic, aiming to restore mobility and alleviate pain [2]. The success of this procedure, however, is influenced not only by the implant but also by several surgical parameters, most notably the surgical approach [3,4,5,6]. The choice of hip approach has been the subject of longstanding debate among orthopedic surgeons [7,8]. Each approach presents distinct anatomical considerations that may affect post-operative stability, functional recovery, and the risk of approach-specific complications [4,9]. Among the most employed techniques for femoral HA, the postero-lateral (PL) and direct lateral (DL) approaches remain two of the principal options in current orthopedic practice [10]. The PL approach, known for its extensive exposure, has historically been associated with higher dislocation rates [11], whereas the DL approach, often regarded for its potentially lower dislocation risk due to soft tissue repair, has been scrutinized for possible effects on abductor muscle function and gait abnormalities [9,12,13]. Despite their widespread use, the optimal surgical approach for HA in the context of FNFs remains a matter of debate, with no clear consensus to date [14]. Recent systematic reviews and meta-analyses have compared multiple hip approaches simultaneously, inevitably introducing heterogeneity and limiting interpretability [3,4,6,7,10]. A recent 2024 meta-analysis, although comprehensive in its evaluation of four approaches, reported no significant differences in most objective outcomes but found substantial variability in functional and pain-related measures [5]. Moreover, the relatively short follow-up durations in these broader studies limit conclusions regarding long-term outcomes, complication profiles, and durability of the surgical results. Therefore, this systematic review and meta-analysis aims to provide a focused long-term comparison of the PL and DL approaches for femoral HA in patients with FNFs. By synthesizing only studies that directly compare these two techniques and requiring a minimum follow-up of one year, we seek to clarify enduring differences in operative time, dislocation incidence, infections, perioperative fractures, and reoperations. In addition, we evaluate long-term patient-reported outcomes (PROMs), including quality of life (EQ-5D), pain levels (VAS), and overall satisfaction. Our goal is to offer clearer, evidence-based guidance to orthopedic surgeons in selecting the most appropriate surgical approach, ultimately improving patient outcomes following this commonly performed procedure.

## 2. Materials and Methods

This systematic review and meta-analysis were conducted in accordance with the Preferred Reporting Items for Systematic Reviews and Meta-Analyses (PRISMA) guidelines. The completed PRISMA 2020 checklist is provided in the Supplementary Materials (File S1).

### 2.1. Eligibility Criteria

Only comparative studies written in English meeting the following Population, Intervention, Comparison, and Outcome (PICO) criteria with a minimum follow-up period of 1 year were included:

- P (Population): Adults with femoral neck fractures (FNFs) treated with hemiarthroplasty (HA). Only acute, traumatic FNFs were eligible. We excluded patients with pathological fractures, bilateral procedures, revision HA, or non-traumatic indications
- I (Intervention): HA performed via a PL surgical approach.
- C (Comparison): HA performed via a DL surgical approach.
- O (Outcomes): The primary outcomes of interest include operative time, incidence of dislocations, infections, perioperative fractures and reoperations. Secondary outcomes include PROs, specifically quality of life (EQ-5D scores), pain levels (VAS scores), and overall patient satisfaction. Studies must report at least one of these specified outcomes.

We excluded non-comparative designs such as case reports, case series, conference abstracts, narrative or systematic reviews, editorials, letters to the editor, expert opinions, and biomechanical, cadaveric, or animal studies. We also excluded studies evaluating hemiarthroplasty for degenerative conditions, revision surgery, or fracture types other than femoral neck fractures (e.g., intertrochanteric or subtrochanteric fractures). Studies involving pathological fractures, bilateral procedures, or non-traumatic indications were not eligible. Furthermore, studies that did not directly compare the postero-lateral and direct lateral approaches were excluded. When duplicate publications or multiple articles based on the same patient cohort were identified, only the most complete or methodologically robust version was retained for analysis.

### 2.2. Search Strategy

A comprehensive search was performed across three major electronic databases: PubMed, Scopus, Web of Science and Cochrane Library. The following search string was consistently applied to both databases: (“hemiarthroplasty” OR “hip hemiarthroplasty” OR “femoral head prosthesis” OR endoprosthesis OR “partial hip replacement”) AND (“posterior approach” OR “posterolateral approach” OR “postero-lateral approach”) AND (“direct lateral approach” OR “lateral approach” OR “transgluteal approach” OR “Hardinge approach”). The search covered all years up to the date of execution to capture all relevant published literature.

### 2.3. Study Selection

Duplicate records were meticulously removed. Two independent reviewers (GM and FRP) screened the titles and abstracts for eligibility against the predefined inclusion and exclusion criteria. Any studies deemed potentially relevant or where eligibility was uncertain proceeded to full-text review. In the second phase, the reviewer independently assessed the full-text articles.

### 2.4. Data Extraction

A standardized, pre-piloted data extraction form, designed using Microsoft Excel, was used to systematically extract relevant information from each included study. Data extraction was performed by one reviewer (GM) and verified by a second reviewer (FRP). The following data points were extracted: study characteristics (first author, year of publication, study design, number of participants in each arm), patient demographics (mean age, sex distribution), intervention details (specifics of the PL and DL approaches and duration of follow-up), and outcome data (operative time, dislocations, infections, perioperative fractures, reoperations, EQ-5D, VAS scores, patient satisfaction). Data at comparable follow-up points (e.g., 12 months and latest follow-up) will be prioritized.

### 2.5. Risk of Bias Assessment

The methodological quality and risk of bias of the included studies were independently assessed by one reviewer (GM). Discrepancies were resolved through discussion with a second reviewer (FRP). For Randomized Controlled Trials, the Cochrane Risk of Bias tool 2.0 was used. Bias was evaluated across five domains: randomization process, deviations from intended interventions, missing outcome data, outcome measurement, and selection of the reported result. For each domain, the level of bias was judged as “low risk,” “some concerns,” or “high risk”. For prospective and retrospective studies, the methodological quality was assessed with the MINORS criteria. This tool assesses studies based on 12 items: clearly stated aim, inclusion of consecutive patients, prospective collection of data, endpoints appropriate to the study aim, unbiased assessment of the study endpoint, follow-up period appropriate to the study aim, loss to follow-up less than 5%, prospective calculation of the study size, adequate control group, contemporary groups, baseline equivalence of groups, and adequate statistical analyses. Each item is scored from 0 (not reported) to 2 (reported and adequately performed), with a maximum score of 24. Studies were categorized based on their total MINORS score, indicating “low risk,” “moderate risk,” or “high risk” of bias.

### 2.6. Data Synthesis

A systematic review of the findings is presented for all included studies, summarizing their characteristics and key results. Furthermore, a meta-analysis was performed for the primary objective outcomes (operative time, dislocations, infections, perioperative fractures, and reoperations) using Review Manager (RevMan) software version 5.4. For dichotomous outcomes (dislocations, infections, fractures, and reoperations), odds ratios (OR) with 95% confidence intervals (CI) were calculated. For continuous outcomes (operative time), mean differences (MD) with 95% CI were calculated.

## 3. Results

**Figure 1:**
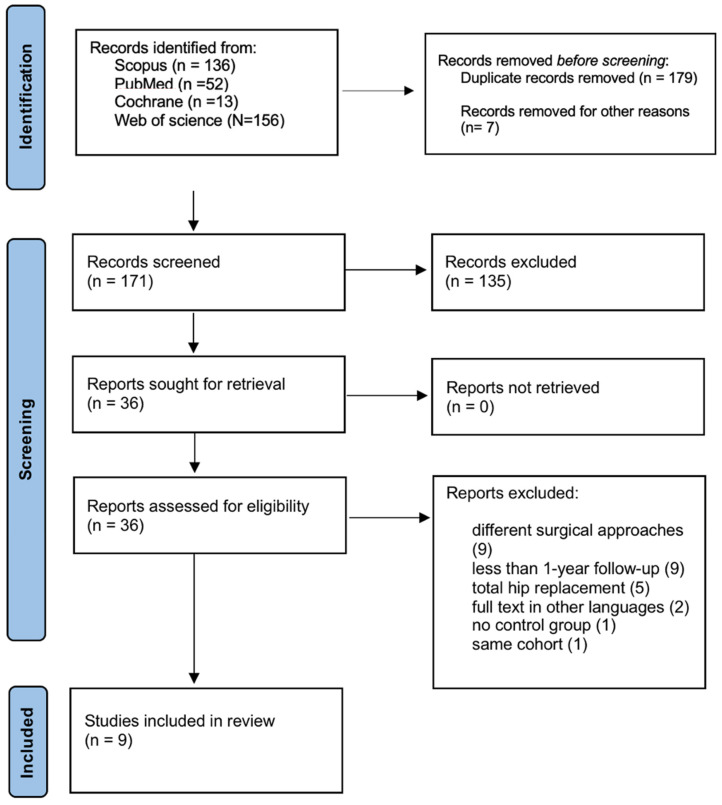
PRISMA flow diagram.

Our comprehensive search across PubMed, Web of Science, Scopus and Cochrane Library yielded a total of 357 unique records. Following the removal of 186 duplicates, 171 titles and abstracts were screened for eligibility. Of these, 36 full-text articles were retrieved for detailed assessment. After thorough review, 9 studies met our predefined inclusion criteria and were included in the final qualitative synthesis and meta-analysis where applicable. A PRISMA flow diagram (Figure 1) details the selection process.

### 3.1. Characteristics of Included Studies

The nine included studies, published between 2014 and 2021, comprised one Randomized Controlled Trial (RCT), two prospective cohort studies, and six retrospective studies, encompassing a large total cohort of 82,076 patients. Of these, 43,802 patients underwent HA via the PL approach, and 38,274 received the DL approach. The median follow-up period across all studies was 12 months, though some large registry studies provided longer-term data. While patient demographics (mean age, sex distribution) were generally comparable between the PL and DL groups within most individual studies, significant baseline differences in factors crucial for dislocation risk were noted in some cohorts. These differences primarily included the type of implant used (unipolar vs. bipolar [15], cemented vs. uncemented [9]) and the patient’s preoperative health status (ASA class) [15]. A detailed breakdown of study design, cohort size, and patient characteristics is provided in Table 1.

### 3.2. Risk of Bias Assessment

The methodological quality and risk of bias of the included studies varied significantly. The only RCT (Parker, 2015) was terminated early; for this RCT, the risk of bias was assessed using the RoB 2.0 tool, resulting in some concerns (Table 2) [14].

All other studies were observational and acknowledged their intrinsic methodological limits. The mean MINORS score for prospective and retrospective studies was 17 ± 4.3, indicating significant methodological imperfections (Table 3).

### 3.3. Risk of Bias Assessment

**Figure 2:**
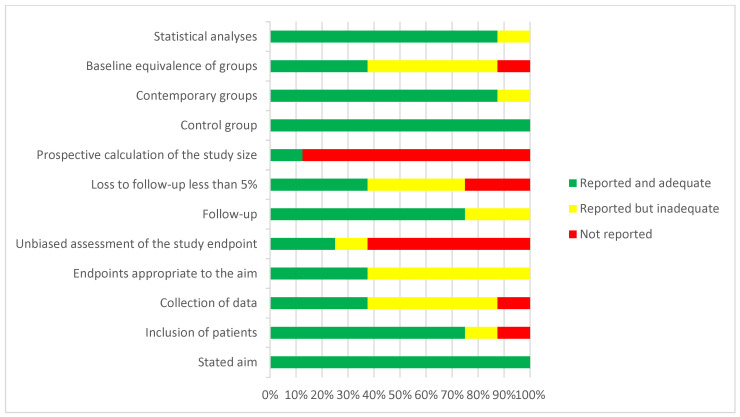
A visual representation of the overall MINORS scores for each methodological aspect of every study considered.

The most common issues, as displayed in Figure 2, included the lack of a prospective sample size calculation in nearly 90% of studies, which questions their statistical power. Furthermore, over half of the studies had issues with the blinding of outcome assessors, leading to a high risk of detection bias. Approximately half showed concerns regarding prospective data collection and baseline equivalence, while one-fifth reported loss to follow-up exceeding 5%, which risks selection bias. In summary, the available data are often limited by deficiencies in statistical planning, objectivity of outcome measurement, and follow-up management—crucial aspects when interpreting these studies’ results.

### 3.4. Operative Outcomes

#### Operative Time

Data on operative time were reported by 4 studies. Kristensen et al. specifically reported a shorter mean operative duration in the postero-lateral group (67 min) compared to the lateral group (76 min), a statistically significant difference [9]. Mukka et al., conversely, noted that surgical time was longer in the DL approach, attributing it to the time spent re-attaching the gluteus medius muscle [12]. Parker did not find a significant difference in operative time, though the postero-lateral approach was subjectively felt to be slightly more difficult by the surgeon [14]. This indicates a mixed picture, with some studies suggesting potential efficiency advantages for one approach, while others find comparable or even slightly longer times for the DL approach depending on specific techniques (Table 4).

**Figure 3:**
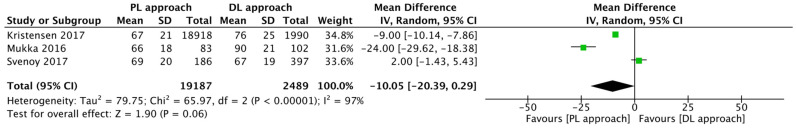
Forest plot comparing the effect of the PL approach versus the DL approach on operative time [9,12,16].

Only 3 studies could be included in the meta-analysis because of the lack of data on standard deviation in the study by Parker et al. The meta-analysis indicated that the mean operation time was inferior in the PL group (shorter), but without reaching statistical significance (Tau^2^ = 79.75; I^2^ = 97%; MD = −10.05 min, 95% CI: −20.39 to 0.29; I^2^ = 97%; *p* = 0.06) (Figure 3).

### 3.5. Post-Operative Complications

#### 3.5.1. Dislocations

Dislocation rates were a frequently reported outcome, with a clear trend emerging. Hongisto et al. observed dislocations in four (3.4%) patients in the postero-lateral approach group, compared with no patients in the lateral approach group [13]. Jobory et al., in a large registry-based study (over 25,000 patients), provided robust evidence, demonstrating a significantly higher dislocation rate for the postero-lateral approach (7.2%) compared to the direct lateral approach (2.7%). They identified the postero-lateral approach as the most pronounced risk factor for dislocation (OR = 2.7; CI 2.3–3.1) [18]. Leonardsson et al. similarly found dislocation to be twice as common in the postero-lateral group (2%, n = 20) compared to the direct lateral group (0.9%, n = 10; *p* = 0.02) among re-operated cases [15]. Rogmark et al., analyzing over 33,000 patients, also found that the postero-lateral approach clearly increased the risk of reoperation due to dislocation [11]. Svenøy et al. found a significantly higher risk for prosthetic dislocation in the postero-lateral group (8%) compared to the lateral approach (1%), with multivariate logistic regression identifying the postero-lateral surgical approach as the only statistically significant predictor of dislocation (OR 8.5, *p* < 0.001) [16]. In contrast, Mansouri et al. reported 6 dislocations (6.1%) in the direct lateral approach and 1 (1.81%) in the postero-lateral approach but found no significant difference (*p* > 0.05) in their smaller cohort [17]. Parker reported only one dislocation in the postero-lateral group, noting that the study’s size was too small to draw conclusions on infrequent surgical complications [14]. Overall, the preponderance of evidence, especially from large registries and Svenøy et al., indicates a higher dislocation risk with the postero-lateral approach (Table 5).

**Figure 4:**
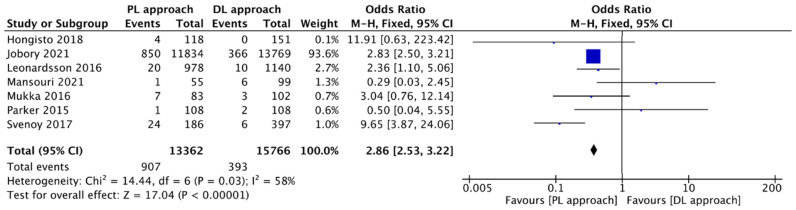
Forest plot comparing the effect of the PL approach versus the DL approach on the risk of dislocation [12,13,14,15,16,17,18].

The meta-analysis, encompassing 7 studies, found a statistically significant difference in the rate of dislocation in favor of the DL group compared to the PL group (I^2^ = 58%; OR = 2.86, 95% CI: 2.53 to 3.22; *p* < 0.00001). This pooled quantitative finding definitively confirms that the PL approach is associated with a nearly three-fold higher risk of post-operative dislocation compared to the DL approach (Figure 4).

#### 3.5.2. Infections

Information on infection rates was available from 5 studies. Mansouri et al. reported no significant difference in joint infection rates between the two techniques (*p* > 0.05) [17]. Leonardsson et al. found infections to be an equally common reason in both direct lateral and postero-lateral groups for reoperation [15]. Rogmark et al. noted an increased risk of reoperation due to infection in younger patients and with uncemented stems but did not specifically link it to surgical approach [11]. Parker also found no significant difference in general medical complications, including infections, between the two approaches [14]. Svenøy et al. reported a higher infection rate in the postero-lateral group (6%) vs. the lateral group (5%), though they noted this was higher than some previous studies and could be influenced by patient selection in other trials [16]. Most studies, including Mukka et al., indicated generally low rates with no significant difference between the PL and DL approaches regarding infection [12] (Table 5).

**Figure 5:**
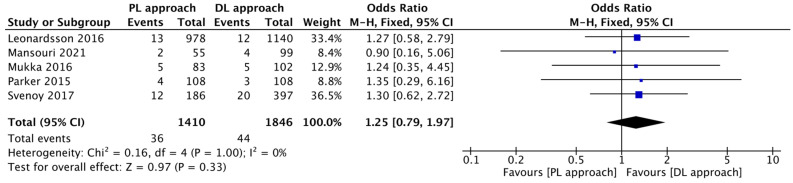
Forest plot comparing the effect of the PL approach versus the DL approach on the risk of infection [12,14,15,16,17].

The rate of infection was shown to be lower in the DL group, but this difference was not statistically significant (I^2^ = 0%; OR = 1.25, 95% CI: 0.79 to 1.97; *p* = 0.33) (Figure 5).

### 3.6. Perioperative Fractures

Data on perioperative fractures were reported by 4 studies. Rogmark et al. found an increased risk of reoperation in the postero-lateral approach due to periprosthetic fracture [11]. Parker noted a tendency for more later fractures of the femur in the postero-lateral group (four cases vs. one in lateral), though the study was underpowered for such infrequent events [14]. Svenøy et al. found no differences between the groups regarding post-operative periprosthetic fractures [16]. The incidence was generally low for both approaches across the included studies, with no consistent evidence suggesting a statistically significant difference between the PL and DL techniques, beyond the trend observed by Rogmark et al. [11]. Mansouri et al. found no significant difference regarding perioperative fractures [17] (Table 5).

**Figure 6:**
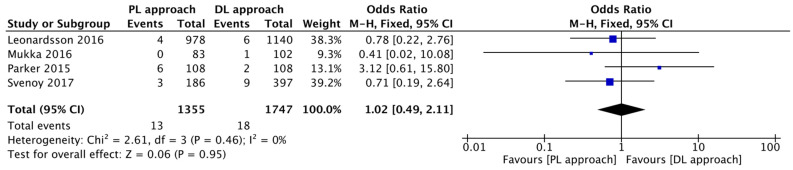
Forest plot comparing the effect of the PL approach versus the DL approach on the risk of perioperative fractures [12,14,15,16].

The rate of perioperative fracture was similar between the two groups, showing no statistically significant difference (I^2^ = 0%; OR = 1.02, 95% CI: 0.49 to 2.11; *p* = 0.95) (Figure 6).

### 3.7. Reoperations

The need for reoperations was assessed in 5 studies. Kristensen et al. reported more reoperations after the postero-lateral approach (8-year survival of 93%) than after the direct lateral approach (8-year survival of 96%), though the risk of reoperation was statistically similar in the first 8 years regardless of the original approach (RR = 1.2, 95% CI: 0.92–1.4; *p* = 0.2) [9]. Jobory et al. reported an overall HA revision rate due to dislocation of 1.6%, with 2.0% for the postero-lateral approach and 1.2% for the direct lateral approach (*p* < 0.001) [18]. Leonardsson et al. found that dislocation was a principal reason for reoperation, being twice as common in the postero-lateral group [15]. Mansouri et al. reported no significant difference in repeated surgery rates [17]. Mukka et al. observed that 12.9% of hips required reoperation, with a lower rate in the DL group (8.8%) compared to the PL group (18.1%), showing a tendency towards statistical significance (adjusted OR 0.42; 95% Cl, 0.16 to 1.11; *p* = 0.08) [12]. Rogmark et al. concluded that the lateral approach helps prevent reoperations associated with dislocation [11]. Svenøy et al. highlighted that more than half the dislocations in their material became recurrent, leading to poor results after treatment of the second dislocation, and that 6/10 patients with recurrent dislocations needed more than one revision procedure [16]. This suggests a potential trend towards lower reoperation rates with the direct lateral approach, often driven by reduced dislocation risk, although not consistently statistically significant across all studies, especially when considering different implant types (Table 5).

**Figure 7:**
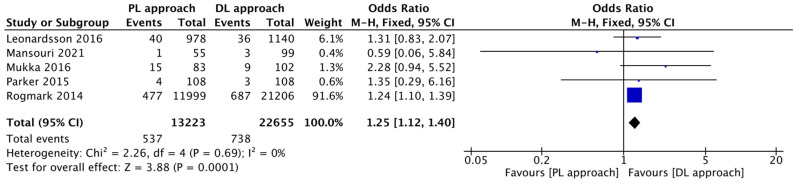
Forest plot comparing the effect of the PL approach versus the DL approach on the risk of reoperation [2,12,14,15,17].

Our meta-analysis showed a statistically significant difference in favor of the DL group compared to the PL group (I^2^ = 0%; OR = 1.25, 95% CI: 1.12 to 1.40; *p* = 0.0001). This quantitative result confirms that the increased risk of reoperation for the PL approach is directly attributable to the higher risk of dislocation, supporting findings from registry studies. The severe consequences of recurrent dislocations, highlighted by Svenøy et al., further underscore the clinical importance of this difference (Figure 7) [16].

### 3.8. Patient-Reported Outcomes

PROMs were variably reported across the included studies, with important differences in measurement tools, timing of assessment, and statistical adjustment. For clarity, the findings are summarized according to the three outcomes of interest—EQ-5D, VAS, and patient satisfaction—and detailed in Table 6.

#### 3.8.1. EQ-5D

Regarding quality of life, only two studies provided EQ-5D scores at one year. Kristensen et al., in their large registry study, reported significantly higher EQ-5D values for the postero-lateral approach at 12 months [9]. However, Leonardsson et al.’s However, Leonardsson et al. found that their initially favorable crude estimates for the PL approach lost significance after adjustment for age, cognitive impairment, and ASA class, suggesting that the apparent advantage was partly confounded by baseline differences [15]. Additionally, another study that we did not consider in this analysis, Svenøy et al., highlighted that recurrent dislocations, more frequent with the PL approach, have a potential negative impact on quality of life [16,17] (Table 6).

#### 3.8.2. Pain

Pain outcomes also showed marked variability. Kristensen et al. reported less pain in the PL group [9], whereas Mukka et al. [12]. The initially favorable unadjusted results observed in Leonardsson et al. [15] were not maintained after full adjustment for confounding variables. Taken together, the available evidence does not suggest a consistent difference in pain levels between the two surgical approaches (Table 6) [13,15].

#### 3.8.3. Satisfaction

Satisfaction outcomes were reported in two studies. Kristensen et al. described better satisfaction in the PL group, but this was not confirmed by Leonardsson et al., whose adjusted analysis eliminated the initial crude association (Table 6) [9,15].

### 3.9. Other Notable Findings

Supporting a functional disadvantage for the lateral approach, Kristensen et al. found that patients reported statistically significantly more walking problems (EQ-5D mobility dimension) in the DL group than in the PL group at all post-operative follow-ups [9]. Jobory et al. and Svenøy et al. also highlighted the issue of Trendelenburg gait/limp as being more frequent with the lateral approach, suggesting a residual effect on hip abductor function, despite these studies finding no difference in standard functional scores (like HHS). Hongisto et al. reported that, at one-year post-hip fracture, patients undergoing surgery via the lateral approach demonstrated a higher need for ambulatory aids compared to those treated with the postero-lateral approach (12% vs. 22% able to ambulate without aids, *p* = 0.026). Multivariate logistic regression identified the lateral approach as an independent risk factor for the need for ambulatory aids or immobility (OR = 2.73, 95% CI = 1.15–6.50). This study also found no statistically significant effect of surgical approach on 1-year survival [13]. Mukka et al. found no significant difference between the two approaches regarding WOMAC or HHS scores but did note a high mortality rate (39.3%) regardless of surgical approach, with no statistically significant difference between groups [12]. Parker confirmed no significant differences in mobility scores or mortality rates between the groups [14]. Rogmark et al. showed that younger patients (60–74 years) had an inferior outcome after HA compared to older patients, with a higher risk of reoperation due to dislocation, infection, and erosion [11]. Svenøy et al. found no differences between groups regarding mortality. They also reported on the severe consequences of multiple dislocations, with many patients needing multiple revision procedures or experiencing complications like deep infections and ultimately poor results [16].

## 4. Discussion

This systematic review and meta-analysis rigorously compared the PL and DL approaches for HA in FNFs, integrating a robust quantitative synthesis for five objective outcomes with a qualitative review of patient-reported data. Our findings present a nuanced picture, with some outcomes showing consistency across studies while others highlight significant discrepancies. Regarding operative time, the data remains varied: while some studies (Kristensen et al., Mukka et al.) indicated differences influenced by technical steps like muscle re-attachment, the rigorous RCT by Parker found no significant difference, suggesting that surgeon familiarity and specific institutional protocols are key determinants of operative duration rather than the approach itself [9,12,14]. However, a strong and consistent signal emerged concerning stability and reoperations. Multiple large registry analyses (Jobory et al., Leonardsson et al., Rogmark et al.) and the study by Svenøy et al. strongly demonstrated a significantly higher risk of dislocation, and consequently an increased risk of reoperation for instability, with the postero-lateral approach compared to the direct lateral approach [11,15,16,18]. This robust evidence highlights the superior stability of the direct lateral approach, establishing it as the safer option for preventing the most common and damaging mechanical complication. Although overall infection and perioperative fracture rates were low and generally comparable, the higher overall reoperation rates in the postero-lateral group were attributed mainly to increased instability risk. Crucially, the studies noted that reoperation rates alone may underestimate the actual burden of this complication, as many dislocations are managed non-surgically, yet recurrent dislocations often lead to poor patient outcomes and further complex revision surgeries. The meta-analysis provides definitive quantitative evidence for the critical difference between the two approaches: the direct lateral approach offers superior mechanical stability. The pooled data definitively establishes a significantly lower risk of dislocation (OR = 2.86) and a significantly lower risk of reoperation for the DL approach. This finding validates the strong signal previously reported in large national registry studies (e.g., Jobory et al., Rogmark et al.), which had the statistical power to detect these relatively infrequent but devastating complications [11,18]. The evidence confirms that the surgical choice has a direct, measurable impact on prosthetic stability. In contrast, the meta-analysis found no statistically significant differences in operative time (*p* = 0.06), infection rates, or perioperative fracture rates. This suggests that the major deciding factor between the approaches should be the risk of instability, as other objective risks are largely comparable, even if the postero-lateral approach might be marginally faster.

Conversely, PROs showed considerable inconsistency. While some studies, notably Kristensen et al., reported better quality of life, less pain, and fewer walking problems with the postero-lateral approach, other high-quality studies like Leonardsson et al. found no statistical association between the surgical approach and PROs (HRQoL, pain, satisfaction) once confounding patient characteristics were rigorously adjusted for [9,15]. This suggests that initial unadjusted benefits favoring the postero-lateral approach may be primarily due to baseline patient differences. Furthermore, the noted association between the lateral approach and a greater incidence of walking problems suggests a potential trade-off that surgeons must consider: improved mechanical stability versus the potential, though inconsistent, impact on abductor function.

## 5. Limitations and Conclusions

Despite providing a focused synthesis and a robust meta-analysis, this review is limited by a scarcity of high-quality RCTs. It relies heavily on observational studies, which are inherently biased and may introduce residual confounding. Varying follow-up periods and the common failure of registries to capture all non-revised dislocations further complicate the ability to draw definitive long-term conclusions. Additionally, due to the limited number of studies available for each pooled outcome, a formal sensitivity analysis could not be performed, as it would have yielded statistically unstable and potentially misleading estimates.

In conclusion, the findings of this systematic review and meta-analysis provide strong evidence that the direct lateral approach is consistently associated with a significantly lower risk of post-operative dislocation and reoperations compared to the postero-lateral approach. However, evidence for PROs remains inconsistent. The choice of surgical approach should therefore carefully balance the quantitatively proven lower dislocation risk of the direct lateral approach against potential, though inconsistently reported, advantages in specific PROs for the postero-lateral approach. Future research must prioritize large-scale, well-designed RCTs with standardized, long-term reporting to provide definitive guidance for the subjective outcomes.

## Figures and Tables

**Table 1:** Study characteristics. RCT, randomized controlled trial; PS, prospective study; RS, retrospective study; NR, not reported; SD, standard deviation; LA, direct lateral approach; PA, postero-lateral approach.

Study	Design	Patients per Approach		Follow-Up (Months)	Age	Sex (%F)	Baseline Disparities Detected	Blinding of Outcome Assessment	Risk of Bias
Parker 2015 [14]	RCT	PL DL	108 108	12	84	92	No significant differences.	Yes (Partial), Research nurse blinded for outcome assessment.	Study underpowered (early termination). Performance bias inherent.
Mukka et al., 2016 [12]	PS	PL DL	83 101	12	84.4	70	No significant differences.	Yes (Partial), Research nurse blinded for patient-reported outcomes (PROMs).	High risk of Selection Bias (surgeon’s preference). Partially mitigated by multivariate adjustment.
Svenoy et al., 2017 [16]	PS	PL DL	186 397	12	82.8	74	No significant differences in patient demographics. Source of patients (hospital) and surgical volume differed.	No. Outcome assessment (complication) based on chart review (unblinded).	High risk of Selection/Performance Bias. PL group from one hospital, DL group from three different hospitals.
Kristensen et al., 2017 [9]	RS	PL DL	18,918 7900	12	83	73	Posterior group had more uncemented prostheses and shorter surgery time.	Yes (Objective Data/Partial), PROMs collected susceptible to recall/non-response bias. Reoperation data is objective.	High risk of Selection Bias (observational registry data).
Leonardsson et al., 2016 [15]	RS	PL DL	9781 140	12	85	74	DL group had more unipolar implants and worse health (higher ASA class).	Yes (Partial), PROMs via questionnaire. Reoperation data is objective.	High risk of Selection Bias. Mitigated by multivariate adjustment.
Rogmark et al., 2014 [11]	RS	PL DL	11,523 20,519	32.4 ± 20.4	84	72	Implicit/Not reported. Implants/Approaches not balanced between cohorts.	Yes (Objective Data), Outcome is reoperation (objective, registry-based data).	High risk of Selection Bias (different approach/implants in different hospitals/countries).
Mansouri et al., 2021 [17]	RS	PL DL	55 99	36.70 ± 16.64 36.46 ± 19.48	75.4 78	59	No significant difference.	No. Outcome assessment relied on unblinded review and unblinded phone calls.	Retrospective study on small sample. High risk of Detection Bias (phone calls). High loss to follow-up.
Hongisto et al., 2018 [13]	RS	PL DL	118 151	12	82.8	79	Uncemented stems used more often in the DL group.	No (Implicit), Outcomes assessed by phone interview (unblinded) or via registry/charts.	High risk of Selection Bias (surgeon on duty selected approach).
Jobory et al., 2021 [18]	RS	PL DL	11,384 13,769	12	NR	71	Posterior approach was more common for bipolar HA.	Yes (Objective Data), Outcome is dislocation (closed reduction and reoperation) via cross-registry linkage (objective).	High risk of Selection Bias. Mitigated by extremely large sample and comprehensive adjustment.

**Table 2:** Risk of bias of randomized controlled trial.

		Risk of Bias Domains					
		D1: Randomization Process	D2: Interventions	D3: Missing Outcome Data	D4: Measurement of the Outcome	D5: Selection of Reported	Overall Risk
Study	Parker 2015 [14]	Low Risk	Some Concerns	Low Risk	Low Risk	Low Risk	Some Concerns

**Table 3:** Quality assessment of non-randomized studies (MINORS).

Study	Stated Aim	Inclusion of Patients	Collection of Data	Endpoints Appropriate to the Aim	Unbiased Assessment of the Study Endpoint	Follow-Up	Loss to Follow-Up Less Than 5%	Prospective Calculation of the Study Size	Control Group	Contemporary Groups	Baseline Equivalent of Groups	Statistical Analyses	Total
Mukka et al., 2016 [12]	2	2	2	1	2	2	2	2	2	2	2	2	23
Svenoy et al., 2017 [16]	2	1	2	2	1	2	2	0	2	2	1	2	19
Kristensen et al., 2017 [9]	2	2	1	1	0	2	1	0	2	2	1	2	16
Leonardsson et al., 2016 [15]	2	2	1	1	0	2	1	0	2	2	1	2	16
Rogmark et al., 2014 [11]	2	2	1	1	0	1	0	0	2	2	0	2	13
Mansouri et al., 2021 [17]	2	0	0	2	0	1	0	0	2	1	1	1	10
Hongisto et al., 2018 [13]	2	2	1	1	0	2	1	0	2	2	2	2	17
Jobory et al., 2021 [18]	2	2	2	2	2	2	2	0	2	2	2	2	22

**Table 4:** Operative time across the included studies. SD, standard deviation; NR, not reported.

		Posterior Approach			Lateral Approach		
	Study	Mean	SD	Patients	Mean	SD	Patients
**Operative time**	Mukka et al., 2016 [12]	66	18	83	90	21	101
	Kristensen et al., 2017 [9]	67	21	18,918	76	25	1990
	Svenoy et al., 2017 [16]	69.2	20	186	66.9	19	397
	Parker 2015 [14]	54.0	NR	108	53.6	NR	108

**Table 5:** Post-operative complication.

		Posterior Approach		Lateral Approach	
	Study	Events	Patients	Events	Patients
**Dislocation**	Parker 2015 [14]	1	108	2	108
	Mansouri et al., 2021 [17]	1	55	6	99
	Mukka et al., 2016 [12]	7	83	3	102
	Svenoy et al., 2017 [16]	24	186	6	397
	Leonardsson et al., 2016 [15]	20	978	10	1140
	Hongisto et al., 2018 [13]	4	118	0	151
	Jobory et al., 2021 [18]	850	11,834	366	13,769
**Infection**	Parker 2015 [14]	4	108	3	108
	Mukka et al., 2016 [12]	5	83	5	102
	Svenoy et al., 2017 [16]	12	186	20	397
	Leonardsson et al., 2016 [15]	13	978	12	1140
	Mansouri et al., 2021 [17]	2	55	4	99
**Perioperative fractures**	Parker 2015 [14]	6	108	2	108
	Mukka et al., 2016 [12]	0	82	1	102
	Svenoy et al., 2017 [16]	3	186	8	397
	Leonardsson et al., 2016 [15]	4	978	6	1140
**Reoperation**	Parker 2015 [14]	4	108	3	108
	Mukka et al., 2016 [12]	15	83	9	102
	Leonardsson et al., 2016 [15]	40	978	36	1140
	Rogmark et al., 2014 [11]	477	11,999	687	21,206
	Mansouri et al., 2021 [17]	1	55	3	99

**Table 6:** Patient-reported outcomes.

	Study	Scale Polarity	Posterior Approach	Lateral Approach
**EQ-5D**	Leonardsson et al., 2016 [15]	1.0 = Perfect Health <0.0 = Worse than Death	0.52 ± 0.37	0.47 ± 0.37
	Kristensen et al., 2017 [9]	1.0 = Perfect Health <0.0 = Worse than Death	0.64	0.61
**Pain**	Leonardsson et al., 2016 [15]	0 = No Pain 100 = Worst Pain	17 ± 19	19 ± 20
	Kristensen et al., 2017 [9]	0 = No Pain 100 = Worst Pain	17	20
	Mukka et al., 2016 [12]	0 = No Pain 100 = Worst Pain	20 ± 17	21 ± 22
**satisfaction**	Leonardsson et al., 2016 [15]	0 = Max. Satisfaction 100 = Min. Satisfaction	22 ± 23	24 ± 24
	Kristensen et al., 2017 [9]	0 = Max. Satisfaction 100 = Min. Satisfaction	21	25

## Data Availability

The original contributions presented in this study are included in the article/Supplementary Material. Further inquiries can be directed to the corresponding author.

## Supplementary Materials

The following supporting information can be downloaded at https://www.mdpi.com/article/10.3390/jcm14248846/s1, File S1: PRISMA 2020 Checklist [19].
